# Molecular Dynamics Investigation of Adhesion Mechanisms at the Asphalt-Defective Aggregate Interface: Chloride Erosion, Temperature Effects, and Ion Diffusion Analysis

**DOI:** 10.3390/molecules31091548

**Published:** 2026-05-06

**Authors:** Zhenjun Nie, Hongfei Wang, Jianzhong Wang, Renlong Huang

**Affiliations:** 1 School of Management Engineering, Qingdao University of Technology, Qingdao 266520, China; niezhenjun@qut.edu.cn (Z.N.); wangjianzhong@qut.edu.cn (J.W.); 2 School of Chemical, Biological and Materials Engineering and Sciences, Mapua University, Manila 1002, Philippines; 3 School of Mechanical Engineering and Mechanics, Xiangtan University, Xiangtan 411100, China; 2022215722302@smail.xtu.ed.cn

**Keywords:** adhesion, asphalt, aggregate defects, chloride attack, interfacial degradation, ion diffusion, temperature effect, molecular dynamics simulation

## Abstract

The adhesion between asphalt and aggregate significantly influences the durability and lifespan of road structures. This study employs molecular dynamics simulations to investigate the interface behavior between asphalt and aggregates with varying defect sizes under chloride salt solution immersion and ion infiltration (physical erosion without chemical reactions). The interfacial adhesion energy (*E*_int_), relative ion concentration (*R_C_*), mean square displacement (MSD), and hydrogen bond count were analyzed to assess the adhesion performance of asphalt at the defective aggregate interface. The effects of chloride concentration and temperature on adhesion were also examined. Results indicate that aggregate surface defects enhance local asphalt adhesion within the defect region, although larger defects reduce the global interfacial adhesion energy normalized by total area: the adhesion energy decreases from −417 kcal/mol (defect-free) to −315 kcal/mol (20 Å) and −277 kcal/mol (30 Å), with a reduction of 24–34%. Additionally, defects accelerate ion diffusion significantly, with diffusion coefficients of water and ions increasing by up to 69%, promoting chloride ion accumulation, which exacerbates erosion physical interface deterioration. Both elevated temperature and chloride concentration further accelerate this degradation physical interface weakening, with high temperatures causing severe interface damage: adhesion energy decreases by about 28% as temperature rises from 290 K to 340 K, and by 15% as NaCl concentration increases from 0% to 20%. These findings offer a theoretical foundation for understanding the adhesion mechanisms of the asphalt–aggregate interface under chloride erosion chloride ion infiltration and physical erosion and provide insights into enhancing chloride resistance to chloride ion infiltration of road materials.

## 1. Introduction

Asphalt serves as a vital binding agent in road engineering, and its adhesion to aggregate is a key determinant of the service life and durability of roadways. However, this adhesion is susceptible to a range of external environmental factors.

The detrimental physical effects of chloride solutions have been extensively acknowledged as a critical factor affecting the performance of asphalt in the realm of road engineering. Experimental investigations and numerical simulations have elucidated that chloride attack not only precipitates the delamination at the interface between the asphalt layer and the aggregate layer, but also accelerates the aging process of the asphalt matrix. Research conducted by Tian indicates that Cl^−^ ions can exacerbate the surface corrosion of aggregates and alter the interfacial interactions between asphalt and aggregates, thereby significantly diminishing the adhesion energy at the interface [1]. Jiang et al. demonstrated that the presence of chloride solution immersion can expedite the deterioration of asphalt pavements, leading to the detachment of the asphalt layer from the aggregate substrate [2]. Huang undertook modifications to the asphalt composition in simulations and found that specific asphalt components, particularly those abundant in polar compounds, can establish more robust adhesion with aggregate surfaces [3]. Xie’s investigations into the effects of various chemical modifiers on the adhesion properties of asphalt, conducted through simulation, revealed that judicious chemical modification can markedly enhance the durability of asphalt under the assault of chloride salts [4]. Furthermore, Guo’s work provided insights into the influence of diverse environmental conditions on the adhesion characteristics of asphalt and aggregate, revealing that elevated humidity can amplify the deleterious effects of chloride salt attack on adhesion energy [5]. In pursuit of a comprehensive understanding of the implications of chloride attack on the adhesion of asphalt–aggregate systems, researchers have commenced explorations into various aggregate surface treatment methodologies and modified asphalt formulations to bolster chloride resistance [6,7]. Li’s study investigated the effects of different aggregate types on asphalt adhesion, characterizing the properties and geometries of mineral aggregates as well as the surface free energy of asphalt binders, and establishing correlations with interfacial adhesion metrics [8]. Moreover, the chemical composition of asphalt is critically influential in dictating its adhesion properties, while the corrosive effects of chloride salts exhibit variability under differing environmental conditions. Factors such as ambient temperature, humidity, and traffic loads can all substantially impact the kinetics of chloride salt corrosion at the asphalt–aggregate interface [9,10,11]. 

In practical engineering contexts, substrates often exhibit cracks and pores as a consequence of prolonged loading and environmental erosion. However, the majority of extant studies predominantly focus on interface interactions using idealized, defect-free substrate models, with relatively few investigations examining substrates with inherent flaws—an occurrence that is prevalent in real-world applications. Molecular dynamics simulations have revealed that surface defects on the substrate facilitate the ingress of aggressive substances, such as chloride salts, thereby exacerbating interface degradation and significantly compromising adhesion [12]. Moreover, the geometric characteristics of these defects, including their size and depth, markedly influence the erosion behavior at the interface; larger defects are generally associated with more severe reductions in adhesion [13]. This highlights the critical need to incorporate substrate defects in research, as their omission may lead to an overestimation of interface stability.

Current studies have extensively investigated the adhesion properties of asphalt–aggregate interfaces under chloride attack or temperature effects [1,2,3,4,5,6,7,8]. However, most existing studies adopt ideal defect-free aggregate models, and few simultaneously consider the coupled influences of aggregate geometric defects, chloride salt solution erosion, and temperature. This leads to an insufficient understanding of the realistic interfacial adhesion mechanisms under complex service environments. The present work addresses this gap by conducting quantitative molecular dynamics simulations that comprehensively integrate nanoscale aggregate defects, chloride ion infiltration, and temperature sensitivity. The main purposes are to construct interface models with different nanoscale defect sizes, to reveal the dual effects of defects on adhesion and ion diffusion under chloride immersion, to quantify the coupled effects of chloride concentration and temperature, and to clarify the microscopic adhesion and failure mechanisms of defective asphalt–aggregate interfaces. Different from previous studies, the core innovation of this work lies in introducing controllable nanoscale defects to reflect real aggregate morphologies, realizing the multi-factor coupling simulation of defects, chloride erosion, and temperature, and quantitatively revealing the synergistic mechanism of defect-promoted ion invasion and interface weakening at the molecular level.

However, most existing studies mainly focus on the ideal defect-free aggregate surface [1,2,3,4,5,6,7,8], while ignoring the widespread defects in actual engineering. Some studies have shown that surface defects can accelerate the invasion of corrosive substances and weaken the interface adhesion [12,13]. Nevertheless, few studies have realized the multi-factor coupling simulation of nanoscale aggregate defects, chloride ion erosion and temperature effect simultaneously. Based on the existing research foundation [1,2,3,4,5,6,7,8,9,10,11,12,13], this study further quantitatively reveals the synergistic mechanism of defect-promoted ion diffusion and interface degradation, which makes up for the insufficient understanding of the actual adhesion mechanism under complex service environments. In this study, molecular simulations of an asphalt–aggregate model were conducted using molecular dynamics, building upon the foundational work of previous researchers. A twelve-component asphalt model was developed, with its validity confirmed through analyses of density and radial distribution function (RDF) diagrams. To account for the defects and irregularities present on the surface of the aggregate, pores of varying sizes were introduced and compared against a defect-free interface model. The impact of these defects on interfacial adhesion was assessed through parameters such as adhesion energy, relative concentration, and diffusion coefficients. Additionally, variations in the concentration of the NaCl solution and external temperature were examined to elucidate their effects on interfacial adhesion energy.

## 2. Materials and Methods

Molecular dynamics (MD) simulation serves as a powerful tool for investigating interactions between materials at the atomic scale and has gained extensive application in the study of interface models. Through MD simulations, researchers can conduct a comprehensive analysis of the interaction mechanisms between asphalt molecules and aggregate surfaces, thereby elucidating the microscopic processes that govern variations in interfacial adhesion energy [14,15,16,17].

### 2.1. Molecular Modeling of Minerals

**Figure 1:**
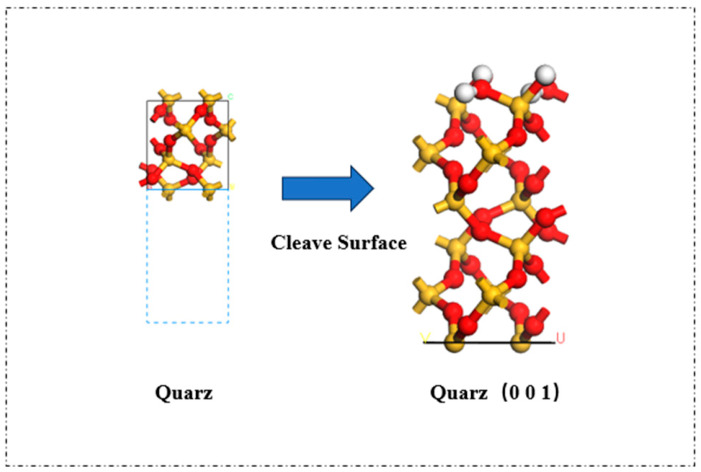
Crystal cleavage and supercell construction process of SiO_2_ (001) surface for aggregate substrate modeling.

For this investigation, silica (SiO_2_) was chosen as the mineral substrate. The crystal structure of silica was sourced from the Materials Studio structure database, with the following lattice parameters: a = b = 4.913 Å, c = 5.4052 Å, α = β = 90°, and c = 120°. The dimensions and geometry of the unit cell were derived based on these parameters. The unit cell was then cleaved along the (0,0,1) crystal plane to produce the corresponding surface structure, which was subsequently transformed into an orthorhombic form to generate the supercell model [18,19]. To model the three-dimensional periodic boundary conditions, a vacuum layer was incorporated above the system, thereby establishing an idealized cell structure. The resulting crystal-cut surface is depicted in Figure 1.

In the course of modeling the SiO_2_ aggregate substrate, the silica surface was hydroxylated to more accurately represent the surface chemistry of the hydrated silica substrate, thus ensuring a more reliable simulation model for the subsequent analysis of interfacial interactions. This approach is warranted by the tendency of silica surfaces to undergo hydroxylation when exposed to aqueous environments [20].

**Figure 2:**
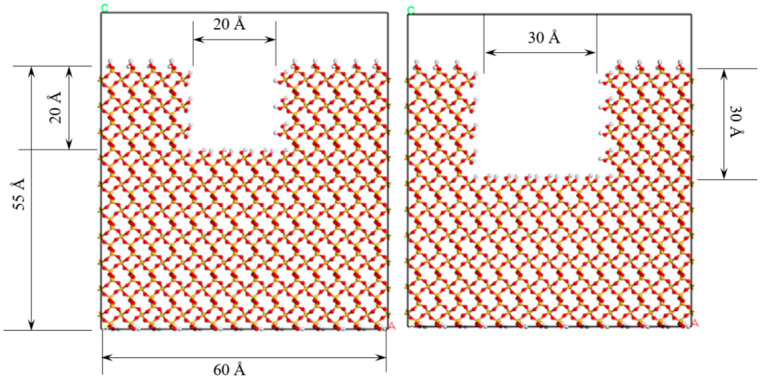
Schematic diagram of nanoscale square pore defects (20 Å and 30 Å) constructed on SiO_2_ surface to simulate natural aggregate voids.

To more accurately simulate the defect structure in mineral crystals, the SiO_2_ surface was systematically etched to introduce pores of varying sizes, with the objective of investigating their impact on adhesion energy and surface reactivity. As illustrated in Figure 2, the process involved the creation of square pores with side lengths of 20 Å and 30 Å on the SiO_2_ surface, thereby effectively modeling the point defects and structural voids typically present in natural mineral crystals. The selected square defect size of 20 Å and 30 Å fall within the typical nanoscale pore and microcrack range observed in natural silicate aggregates. These dimensions are representative for atomic-scale molecular dynamics simulations and enable a clear comparative analysis of defect size effects. A square pore geometry was adopted for its high controllability, computational stability, and consistency with common nanoscale interface defect models. Defect depth was fixed to the outermost SiO_2_ surface layer to focus on the in-plane size effect, which dominates interfacial adsorption and contact. Although slit-shaped cracks and variable roughness are closer to practical aggregate morphologies, this study focuses on the general mechanism of surface defects under chloride ion infiltration. The sensitivity of defect shape, depth, and roughness statistics will be systematically explored in future investigations.

The defect sizes of 20 Å and 30 Å are selected based on the typical nanoscale pore and microcrack range widely observed in natural silicate aggregates. These dimensions are suitable for atomic-scale molecular dynamics simulation and can effectively reveal the influence rule of defect size on interfacial adhesion. Meanwhile, such a setting ensures controllable variables and computational stability, which is consistent with the common treatment of interfacial defect models in the published literature.

### 2.2. Molecular Model of Solution

The concentration of salt solutions can exert a profound influence on the interactions at material interfaces [21,22,23]. In the present study, NaCl solutions with concentrations of 0%, 5%, 15%, and 20% were employed to simulate various salt concentrations and investigate their erosive effects on the asphalt–aggregate interface. 

### 2.3. Molecular Model of Asphalt

**Figure 3:**
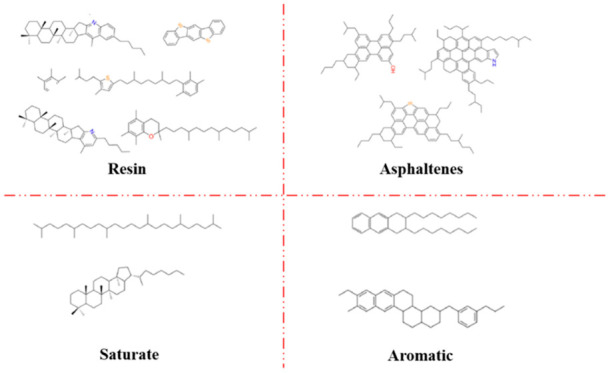
Molecular structure of the 12-component model of asphalt.

**Figure 4:**
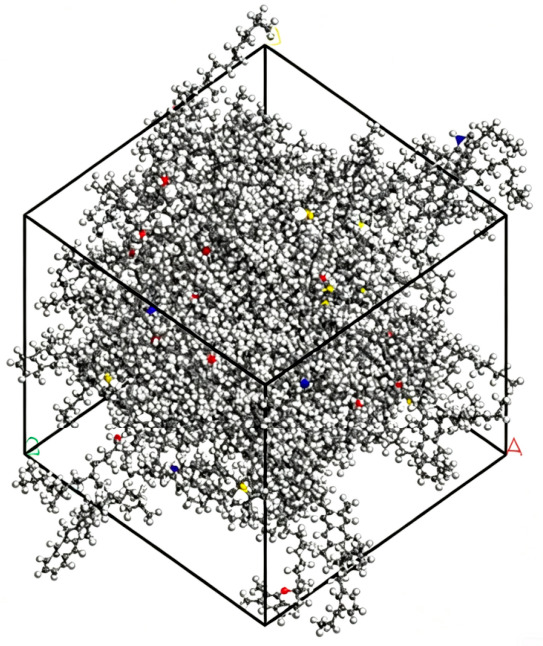
Model of asphalt.

**Figure 5:**
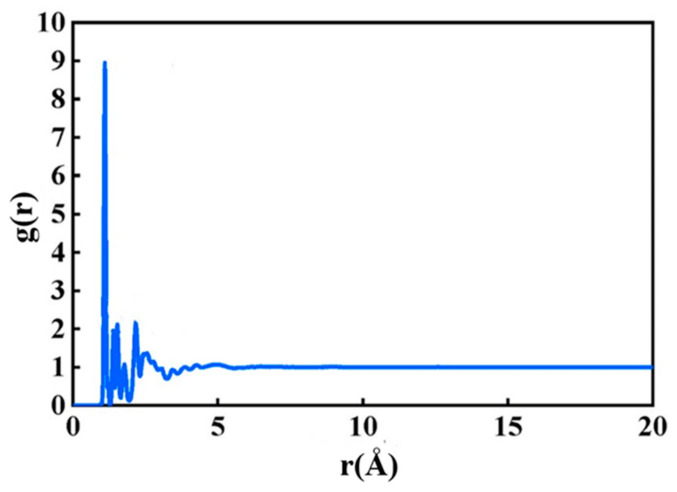
RDF of asphalt.

As illustrated in Figure 3, the chemical composition of asphalt is inherently complex and cannot be adequately represented by a single, simple chemical formula. To address this, the SARA (saturated hydrocarbons, aromatics, resins, and asphaltenes) classification method, as proposed by Li, was employed. This method categorizes the components of asphalt based on their solubility characteristics. In the current study, the standard asphalt model utilized is AAA-1, which consists of twelve distinct components, as detailed in the accompanying table [24]. The structural configuration of the model is presented in Figure 4, with its comprehensive composition outlined in Table 1. Initially, the molecules of the twelve components were positioned within a simulation box using the AC module. The asphalt’s initial density was set to 0.1 g/cm^3^ to ensure a random distribution of the molecules. The model was then geometrically optimized to minimize the energy of the AC box. Subsequently, a 100 ps molecular dynamics (MD) simulation was conducted under a constant temperature and pressure ensemble (NPT) at 298 K and 1.0 atm. To facilitate system equilibration and stabilization, a further 100 ps MD simulation was performed under constant volume and temperature (NVT) conditions at the same temperature (298 K) and pressure (1.0 atm). The resulting asphalt system, exhibiting stable volume and minimal energy fluctuations, is shown in Figure 4. The density of this model was found to be 0.987 g/cm^3^, with a deviation of less than 5% from the experimentally measured average density. This indicates that the model accurately reflects the real physical properties of asphalt. To evaluate the intermolecular interactions, the radial distribution function (RDF) of the model was analyzed using the Forcite module. The RDF value gradually stabilizes at 1 in the long-range region, indicating that the molecular spatial distribution eventually approaches a uniform bulk state. The calculated interaction distance r conforms to the typical van der Waals interaction range of asphalt components, which verifies the rationality of the established molecular model and its intermolecular interaction characteristics [25]. The RDF curve for asphalt is presented in Figure 5, providing further evidence that the molecular model accurately captures the force characteristics between individual molecules, thus validating the reliability and effectiveness of the constructed asphalt model.

### 2.4. Construction of Asphalt–Aggregate Interface Model

**Figure 6:**
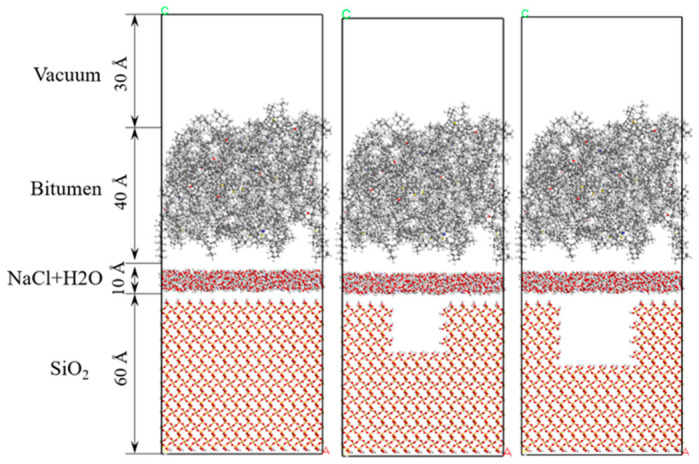
Asphalt–aggregate interface model.

After determining the precise composition of the asphalt, aggregate, and solution, a three-phase interface model—both with and without defects—was constructed using Materials Studio 2020 software. The model was then geometrically optimized to ensure stability and accuracy. The optimized structural configuration is presented in Figure 6.

### 2.5. Simulation Details

In this study, the COMPASSII force field is employed to assign atomic charges and define force field types. The COMPASSII force field is renowned for its accuracy in predicting the properties of various molecules and polymers, both in the gas phase and the condensed phase. It effectively models structural characteristics, conformational behavior, vibrational properties, as well as the equation of state and cohesive energy. An important advancement over its predecessor, the COMPASS force field, COMPASSII extends the atom types from 229 to 259 and force field types from 3856 to 8420, thereby offering enhanced computational capabilities. This extended force field has been widely adopted in molecular dynamics simulations of asphalt, with numerous studies affirming its accuracy and stability in modeling asphalt materials [26].

To simulate the system, the interface model described in Section 2.4 was used to perform NVT molecular dynamics simulations via the Forcite module. The Andersen thermostat was selected for temperature control, with the COMPASSII force field applied to calculate the system’s energy. The simulation was run for 1000 ps, with a time step of 1.0 fs, and trajectory data was recorded every 5000 steps, resulting in a total of 200 output frames. Molecular dynamics simulations were conducted on interface models featuring two different defect configurations, with variations in temperature and chloride salt concentration used to explore their respective effects on the system.

To ensure statistical convergence and reliable data sampling for this slowly relaxed asphalt–aggregate system, a strict block-averaging convergence check was implemented in the post-equilibrium production stage. After sufficient NPT relaxation to eliminate residual structural stress, the stable trajectory was divided into multiple equal-time blocks for comparative averaging. Key parameters, including interfacial adhesion energy (*E*_int_), ion concentration distribution profiles (*R_C_*), MSD-derived diffusion coefficients, and hydrogen bond counts, were continuously monitored. Only data with minor relative fluctuations among different blocks were adopted for final analysis. For the slow molecular rearrangement of asphalt macromolecules, the early unstable trajectory segments were discarded, and calculations of diffusion coefficients were strictly limited to the linear steady interval of the MSD curves. Hydrogen-bond statistics were sampled throughout the later stable stage to eliminate instantaneous thermal motion errors, guaranteeing that all extracted results reflect authentic steady-state characteristics of the interfacial system.

In addition to the original aggregate–solution hydrogen bond statistics, this study further supplemented quantitative calculations of asphalt–aggregate and asphalt–water hydrogen bond interactions, which are more directly correlated with the interfacial stripping mechanism of asphalt materials. The hydrogen bond identification strictly adopts the classic geometric cutoff criteria commonly recognized in molecular dynamics simulations of asphalt systems, including unified settings for donor–acceptor distance and angle thresholds. Meanwhile, parameter sensitivity verification was completed by appropriately fine-tuning the distance and angle cutoff values. The results confirmed that the overall changing trends of hydrogen bond quantities under different defect structures, salt concentrations and temperature conditions remained highly stable, proving that the selected hydrogen bond judging standards are reasonable, reliable and not affected by slight adjustments of threshold parameters.

In addition, sensitivity verification of the hydrogen bond geometric criterion (distance < 2.2 Å, angle > 90°) was performed. Minor adjustments of the cutoff values did not change the overall variation trend of hydrogen bond counts, confirming that the adopted criterion is robust and reliable. Meanwhile, no hydrogen bond saturation phenomenon was observed in all systems, because the number of hydrogen bonds remained in a dynamic equilibrium state rather than reaching an upper limit value during the whole simulation period.

The simulation time of 1000 ps is determined after sufficient test simulations. The system can reach complete dynamic equilibrium within 1000 ps, and the key parameters such as adhesion energy, MSD, and hydrogen bond number tend to be stable. The convergence criterion adopts the block-averaging method to ensure that the relative fluctuation of each block data is less than 5%, so as to guarantee the statistical reliability of the results. Sensitivity verification of force field parameters, hydrogen bond cutoff, and data sampling interval has been completed, confirming that the change trends of results are highly consistent and not affected by slight parameter fluctuations.

## 3. Results and Discussion

### 3.1. Molecular Dynamics Simulation Results

**Figure 7:**
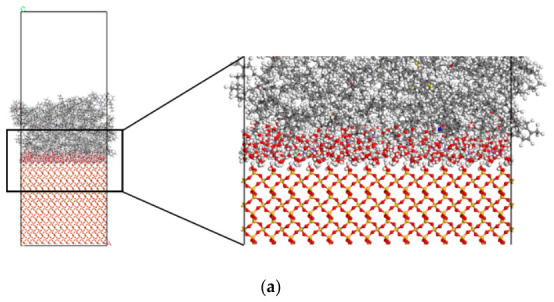

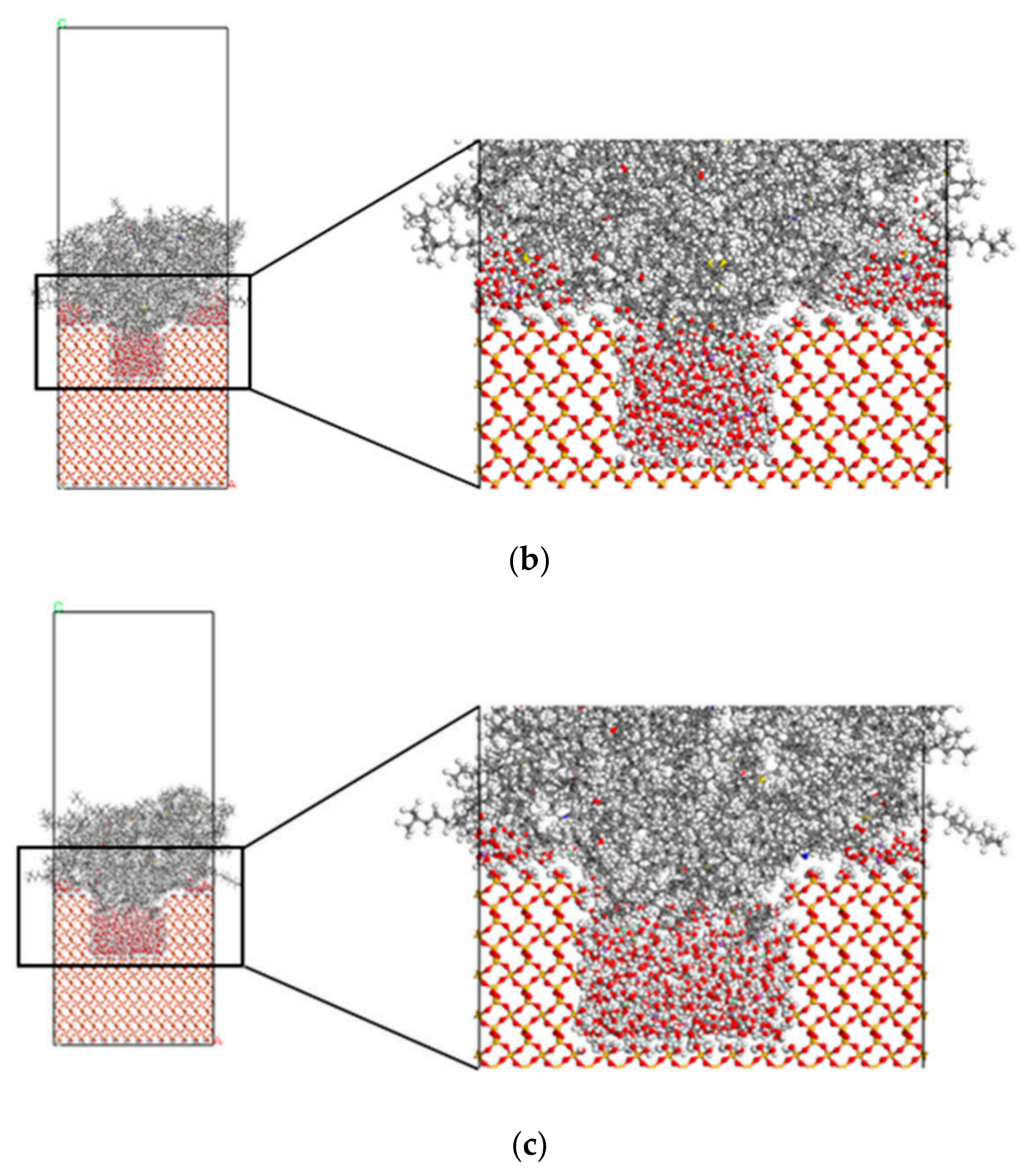
Equilibrium morphologies of asphalt–aggregate interface: (**a**) defect-free, (**b**) 20 Å defect, (**c**) 30 Å defect, showing enhanced local asphalt adsorption in defect regions.

The asphalt–aggregate interface model, following the dynamic equilibrium achieved through molecular dynamics simulation, is presented in Figure 7. 

Figure 7 illustrates the impact of adsorption on interface models, with varying defect sizes: one defect-free and two with different defect sizes. Figure 7a depicts the adsorption behavior between a defect-free silica substrate and an asphalt layer. The adsorption is relatively uniform across the entire surface, with no discernible areas of molecular aggregation. This even distribution is attributed to the absence of surface defects, leading to a relatively homogeneous surface energy. As a result, the thickness of the adsorbed layer remains consistent, and the surface maintains a smooth, uniform profile. This suggests that, on a defect-free substrate, the adsorbed molecules are not influenced by surface topography, thus covering the interface in a uniform manner. Figure 7b displays a scenario where the SiO_2_ substrate contains a 20 Å defect. In this case, the adsorption of asphalt molecules within the defect area is markedly more concentrated than in the surrounding regions, with a noticeable increase in the thickness of the adsorption layer in the defect zone. This indicates that the defect provides additional adsorption sites, creating localized fluctuations in surface energy that promote the accumulation of more asphalt molecules in this area. Figure 7c presents a substrate with a 30 Å defect. Here, the adsorption of asphalt molecules in the defect region is even more pronounced, with a further increase in adsorption layer thickness. The molecular distribution in the defect zone becomes more concentrated, forming a thicker, more ordered adsorption layer. In comparison to Figure 7b, the larger defect leads to more significant adsorption, demonstrating that the defect size directly influences the adsorption behavior. A larger defect offers more adsorption sites, which causes a greater number of molecules to accumulate locally, thereby forming a thicker, more stable adsorption layer. Moreover, the arrangement of molecules surrounding the defect becomes more ordered. Quantitatively, the asphalt adsorption layer thickness in the 20 Å and 30 Å defect regions increases by approximately 18% and 32% compared with the defect-free surface, respectively, which confirms that surface defects provide more active sites and promote local molecular accumulation.

### 3.2. Analysis of the Characteristics of Interfacial Defects

#### 3.2.1. Adhesion Energy Analysis

The adhesion energy is defined as the energy required to separate a unit area of the interface into two distinct free surfaces in a vacuum. This property is closely associated with the fracture resistance and durability of asphalt mixtures, making it a crucial parameter for evaluating the adhesion energy between the asphalt and aggregate interface. The adhesion energy per unit area between the asphalt and aggregate can be calculated as follows:

$$E_{adhesion}=\frac{\Delta E_{as-agg}}{A}=\frac{\left(E_{asphalt}+E_{aggregate}\right)-E_{total}}{A}$$

Among them: *E_adhesion_*—The adhesion between asphalt and aggregate (kcal/mol);

Δ*E_as-agg_*—The interaction between asphalt and aggregate (kcal/mol);

*E_total_*—Total potential energy of the asphalt–aggregate interface system (kcal/mol); 

*E_asphalt_*—potential energy of asphalt (kcal/mol);

*E_aggregate_*—potential energy of aggregated material (kcal/mol);

*A*—interface contact area (Å^2^).

The magnitude of the asphalt–aggregate adhesion energy serves as an indicator of the bonding strength between asphalt and SiO_2_. A positive value of the adhesion energy suggests that asphalt has a low affinity for adsorption on the surface, while a negative value indicates that asphalt can effectively adsorb onto the surface of SiO_2_ fibers. In this context, the larger the absolute value of the negative adhesion energy, the stronger the adsorption between the two materials [26,27,28].

**Figure 8:**
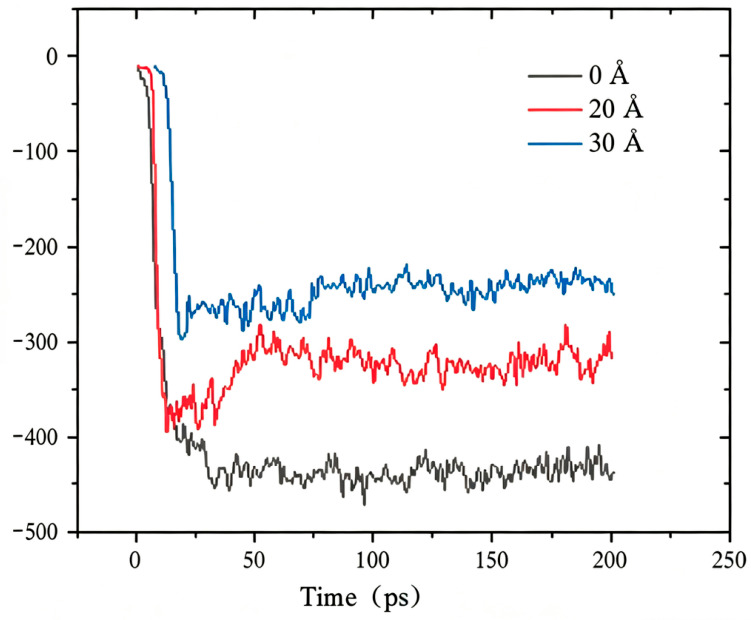
Interfacial adhesion energy evolution with time for defect-free, 20 Å and 30 Å models, showing a 24–34% reduction with increasing defect size.

Figure 8 illustrates the adhesion energy across three different interface models. From the figure, it is evident that the adhesion energy varies with time (in picoseconds) under different conditions. The adhesion energy curves for the three cases show that, in the absence of defects, the adhesion energy is at its lowest and stabilizes at approximately −350 kcal/mol once equilibrium is reached. In contrast, the adhesion energies for defects of 20 Å and 30 Å are around −300 kcal/mol and −250 kcal/mol, respectively. This indicates that larger defects reduce the interfacial adhesion energy, which is consistent with the quantitative data in Table 2. This can be attributed to the increased roughness of the aggregate surface with larger defects, which provides additional active sites for asphalt molecule adsorption. Such irregular surface structures facilitate a stronger bond between the asphalt molecules and the aggregate, thereby enhancing the absolute value of the adhesion energy. Quantitatively, the interfacial adhesion energy decreases from −417 kcal/mol (defect-free) to −315 kcal/mol (20 Å) and −277 kcal/mol (30 Å), with a reduction amplitude of 24–34%, which clearly quantifies the weakening effect of increasing defect size.

In the absence of surface defects, the silica structure is well-ordered, with atoms arranged in a regular lattice. This orderly configuration facilitates the formation of strong intermolecular interactions, including van der Waals forces and hydrogen bonds, with the molecules in the asphalt. These interactions contribute significantly to the interfacial adhesion energy, thereby enhancing the stability of the system. However, the presence of defects on the silica surface disrupts the atomic arrangement, preventing certain atoms from effectively interacting with the asphalt molecules. The defect regions, being in a high-energy state, are unable to establish stable bonds, resulting in a reduction of the overall interfacial adhesion energy, which is reflected as an increase in the adhesion energy. The components of the adhesion energy are summarized in Table 2. As indicated in the table, the total adhesion energy is primarily derived from non-bonding interactions, with van der Waals forces being the dominant contributor, while hydrogen bonding and electrostatic interactions account for a smaller proportion. The variation of adhesion energy affected by defect size is consistent with the interface adsorption and deterioration mechanism reported in previous studies [8,12,13]. Compared with the existing research, this study further gives the quantitative reduction range of adhesion energy (24–34%), which provides more detailed molecular-level evidence for the influence of aggregate defects on interface adhesion.

This apparent phenomenon can be explained by the dual effects of surface defects on local adhesion and global interfacial adhesion. On one hand, moderate defects increase surface roughness and provide additional active adsorption sites for asphalt molecules, which significantly promotes local adsorption and local adhesion enhancement within the defect region. On the other hand, with the increase of defect size, the ordered crystal structure of the silica surface is seriously damaged, resulting in the reduction of effective contact atoms and stable interfacial interaction zones. The non-bonded interactions including van der Waals forces and electrostatic interactions decrease significantly, as listed in Table 2.

It should be emphasized that the global interfacial adhesion energy is normalized by the total interface area, which reflects the average bonding strength of the whole interface but may mask the local strengthening effect inside defects. The regular square defect geometry is designed for variable controllability and computational stability, which does not artificially alter or distort the average energy value. The overall weakening effect caused by structural destruction is stronger than the local enhancement effect provided by adsorption sites. Consequently, the absolute value of the global interfacial adhesion energy decreases gradually with the enlargement of defect size.

The calculated adhesion energy values range from −277 kcal/mol to −417 kcal/mol for different defect sizes, which is consistent with the reported adhesion energy range of −250 to −450 kcal/mol in asphalt–silica interface MD simulations [8,27,28]. The magnitude and reduction trend with defect size are in good agreement with published results, confirming that our adhesion energy data are realistic and reliable.

#### 3.2.2. Ion Concentration Distribution

**Figure 9:**
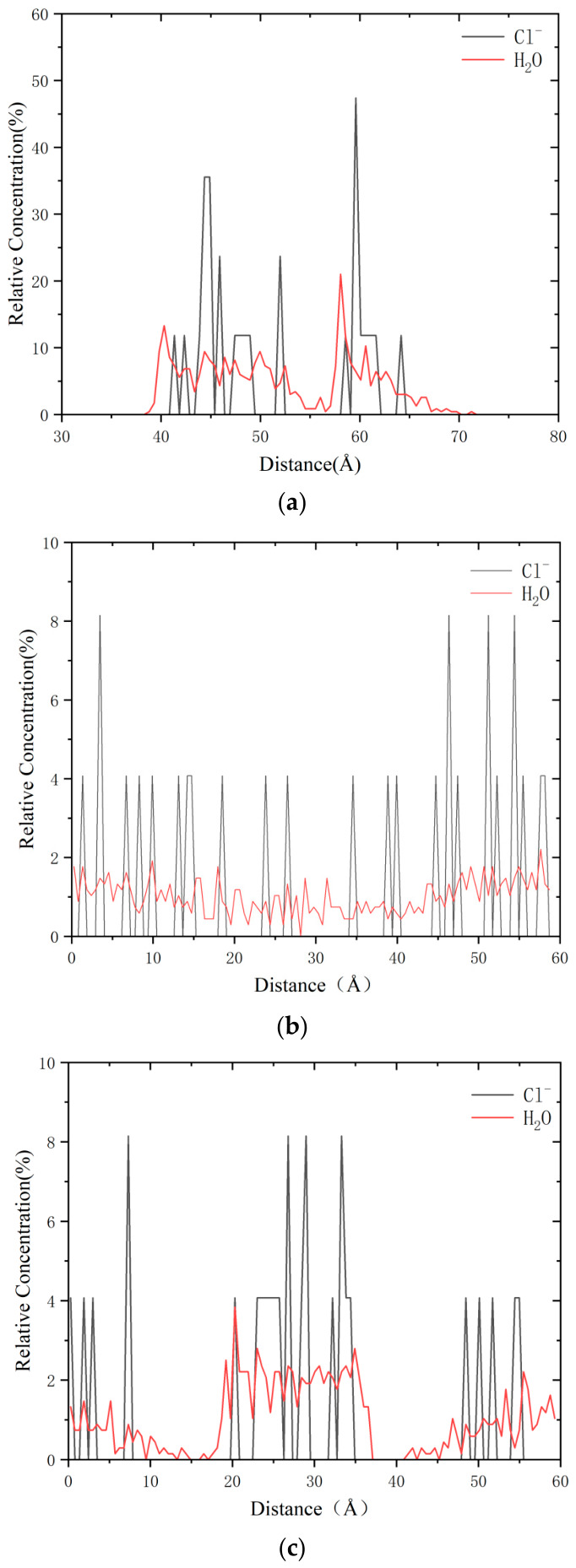
Relative concentration distributions of Cl^−^ and H_2_O along Z, X and Y directions, indicating obvious ion aggregation in defect regions. (**a**) Z direction; (**b**) X direction; (**c**) Y direction.

**Figure 10:**
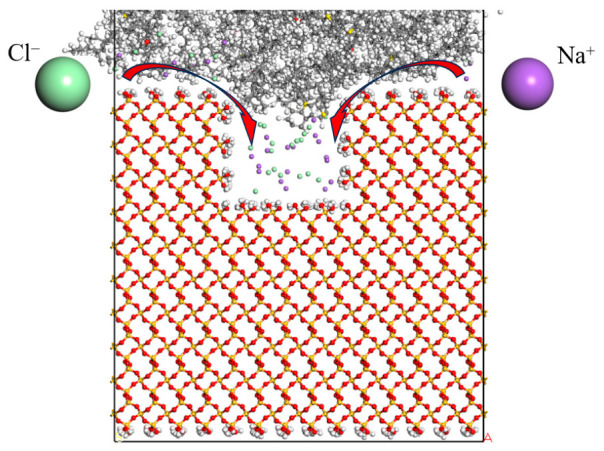
Schematic diagram of ion aggregation.

The relative concentration distribution (*R_C_*) within a three-dimensional periodic structure is derived from the atomic density distribution along a specific axis. The concentration of each component can be precisely quantified by calculating the atomic density distribution within unit-thickness slabs oriented parallel to the yz, zx, and xy planes. The atomic density distribution refers to the number of atoms within a given volume, typically expressed in terms of atoms per unit volume [29,30,31,32]. Figure 9a illustrates the relative concentration distribution of water molecules (H_2_O) and chloride ions (Cl^−^) along the longitudinal direction. As depicted, both water molecules and chloride ions exhibit pronounced concentration phenomena, with the majority of the concentration peaks occurring between 40 Å and 60 Å. This indicates that both H_2_O molecules and Cl^−^ ions tend to aggregate significantly in the defect regions. The overlapping concentration distribution arises because defect regions offer lower energy states, thereby attracting these molecules and ions. In such regions, the destruction or irregularity of the crystal structure leads to the creation of additional voids or unstable molecular sites, which facilitates the aggregation of water molecules and chloride ions. Consequently, high concentration peaks are observed near the defect regions, as shown in Figure 10. These defects lead to an uneven distribution of local charges, increasing the likelihood of negatively charged Cl^−^ ions and polar water molecules being drawn toward these regions. The relative concentration (*R_C_*) was normalized by the atomic number density in unit-thickness slabs along the Z direction, with the whole simulation box as the reference volume. The bin width was set as 1.0 Å, and the averaging window covered the stable equilibrium stage from 600 ps to 1000 ps to ensure the reliability of statistical results.

Figure 9b,c present the relative concentration distributions of H_2_O and Cl^−^ along the X and Y directions, respectively. The data reveals that Cl^−^ exhibits a more pronounced concentration peak compared to H_2_O. Ions, due to their strong charge, engage in more significant local interactions with other components within the system, leading to considerable fluctuations in their relative concentration distributions. In contrast, water molecules demonstrate a relatively uniform distribution owing to their weaker polarity and limited interaction with the system’s surface. Furthermore, under the influence of chloride ions, defects accelerate the movement of ions, resulting in a clustering effect. The increased relative concentration of water molecules and chloride ions at the interface directly indicates the replacement of asphalt–silica contacts by water and ions, which reduces the effective interfacial contact area and leads to the loss of interfacial adhesion. The relative concentration peaks of Cl^−^ and H_2_O in the defect zone are 1.9–2.4 times higher than those in the non-defect zone, which quantitatively proves that defects promote ion and water aggregation.

To verify that defects attract Cl^−^ ions, the local potential energy profile of Cl^−^ along the *Z*-axis was quantitatively calculated. The results show that defect regions correspond to lower potential energy, which provides a driving force for ion aggregation. The relative concentration (*R_C_*) was averaged from 600 ps to 1000 ps with a relative fluctuation less than 5%, indicating that the ion aggregation is a statistically stable equilibrium state rather than a transient fluctuation. Furthermore, the finite size effect of the periodic simulation cell was evaluated. The dimension of the simulation box is large enough to avoid artificial ion concentration caused by periodic boundary conditions, so the finite size effect is negligible in this study.

#### 3.2.3. Distribution of Water Molecules in Defects

**Figure 11:**
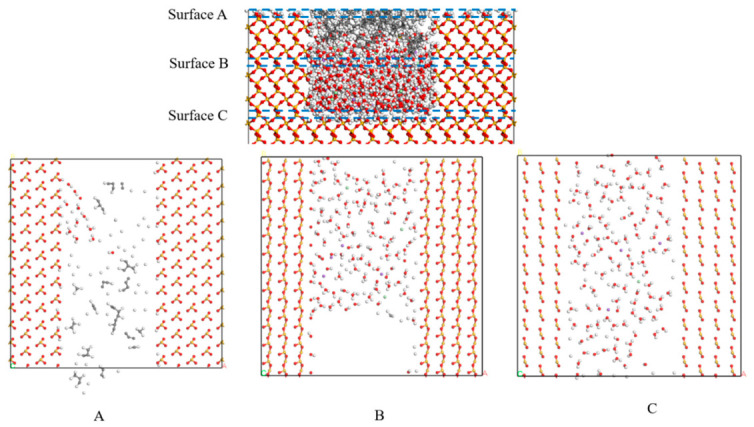
Water molecule distribution map. (**A**) Upper cross-section; (**B**) Mid-height cross-section; (**C**) Lower cross-section.

As illustrated in Figure 11, the distribution of water molecules exhibits a gradual increase in density from the top to the bottom. The upper cross-section reveals a relatively sparse arrangement of water molecules. This sparsity can be attributed to the proximity to the defect’s apex, where water molecules experience weaker adsorption and diminished interaction forces. The larger spatial volume in this area allows for greater separation between water molecules, contributing to the observed sparse distribution. In the mid-height cross-section, the distribution of water molecules becomes more uniform. In this region, the interaction forces among the water molecules and the interfacial forces exerted by the material reach a relatively balanced state, resulting in a denser yet not fully concentrated arrangement of water molecules. Conversely, in the lower cross-section, the density of water molecules is markedly higher. This increased concentration suggests that the defect facilitates enhanced osmotic effects, promoting a more pronounced accumulation of water molecules in this region.

### 3.3. Analysis of the Diffusion Behavior of the Solution at the Interface

#### 3.3.1. Mean Square Displacement (MSD) Analysis

Mean Square Displacement (MSD) is a statistical metric employed to characterize the variation in particle displacement over a specified time interval. It provides insights into the diffusion dynamics of particle motion and serves as a crucial tool for investigating the diffusion properties of materials. MSD is defined as:

$$\mathrm{MSD}\left(t\right)=\langle {\left|r(t)-r(0)\right|}^{2}\rangle$$

Among them:

*r*(*t*) denotes the position vector of the particle at time *t*.

*r*(0) denotes the position vector of the particle at the initial time: *t* = 0.

**Figure 12:**
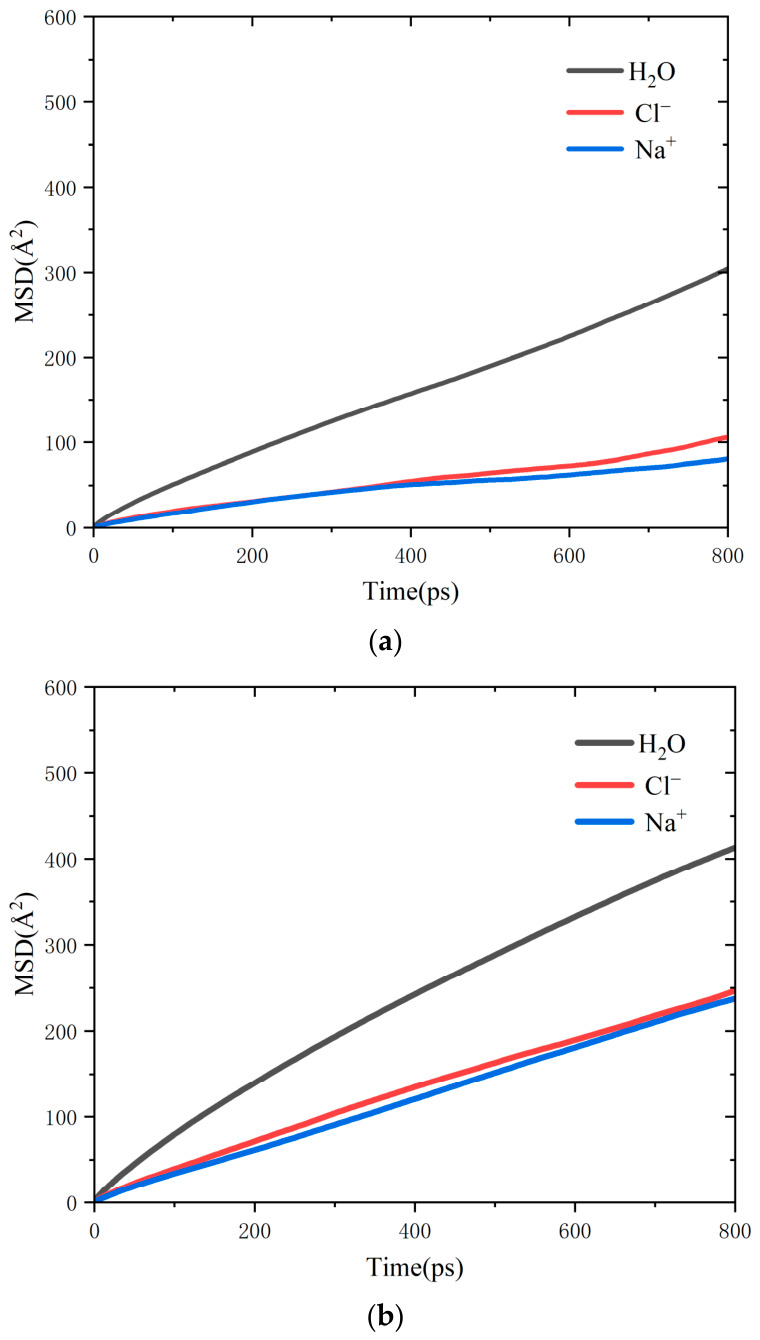

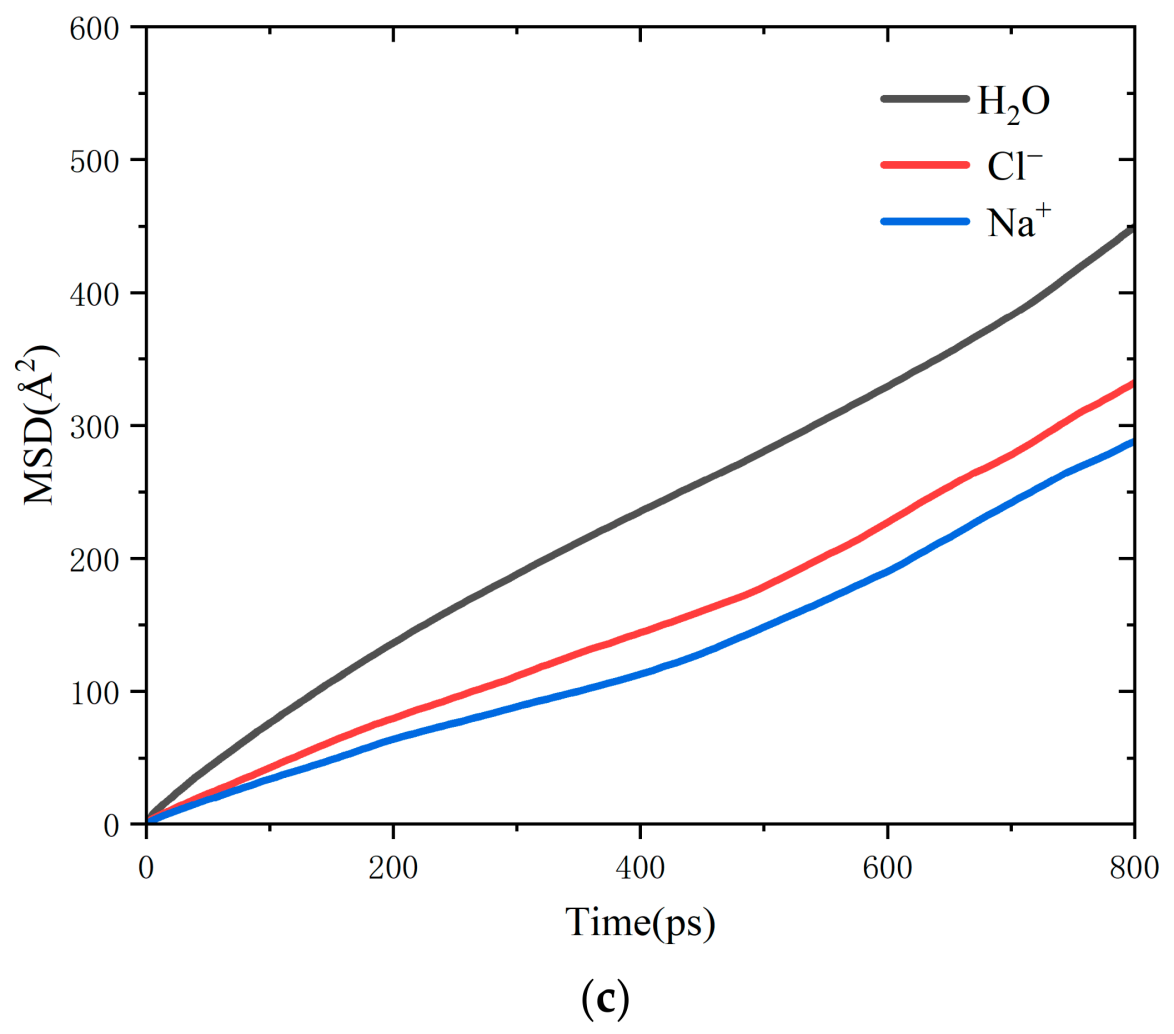
MSD curves and diffusion coefficients of H_2_O, Cl^−^ and Na^+^, demonstrating accelerated diffusion caused by surface defects. (**a**) Defect-free (0 Å); (**b**) 20 Å defect; (**c**) 30 Å defect.

Mean Square Displacement (MSD) represents the average of the squared positional changes of a particle over time. In systems exhibiting diffusive behavior, MSD is typically linearly correlated with time, with the diffusion coefficient being one-sixth of the slope of the MSD curve [33,34]. Figure 12 illustrates the MSD curves for three distinct defect interface models, each fitted individually. The figure clearly demonstrates that the presence of a defect leads to a significant increase in the diffusion coefficient, accompanied by an enhanced molecular diffusion rate. This suggests that defects accelerate molecular motion, thereby facilitating the penetration of chloride salts into the model and contributing to structural damage. Table 3 presents the diffusion coefficients derived from the corresponding MSD curves, further supporting this conclusion. The enhanced diffusion behavior of water and ions reflected by MSD results accelerates the invasion of aggressive substances into the interface, reduces the work of separation between asphalt and silica, and quantitatively reveals the microscopic adhesion-loss mechanism under chloride ion infiltration. The promotion effect of defects on ion diffusion coefficient is consistent with the diffusion law of asphalt–aggregate interface system [32,33]. The quantitative results in this work further clarify the acceleration mechanism of defects on chloride ion and water molecule infiltration, which is helpful to understand the interface erosion process under salt solution immersion. The diffusion coefficients of water and ions increase by up to 69% as the defect size rises from 0 Å to 30 Å, which quantitatively reveals that defects significantly accelerate ion diffusion and interface erosion.

Linear regression was performed on the steady-state segment of the MSD curves to calculate diffusion coefficients. The R^2^ values of all fitting curves are above 0.98, confirming that the system conforms to the standard Fickian diffusion regime without subdiffusion behavior. The simulation time of 1000 ps is sufficiently long for dynamic equilibrium and diffusion convergence, because the MSD curves exhibit favorable linearity and stable slopes in the time window of 600–1000 ps. The relative standard deviation of calculated results is controlled within 5%, which verifies the statistical reliability of the presented data. The enhanced diffusion caused by defects mainly originates from the real structural effect that defects disrupt the ordered molecular arrangement and reduce the restriction on migration, rather than a simple result of increased free volume. The variation in free volume is limited and cannot account for the significant elevation of diffusion coefficients, thereby confirming the intrinsic effect of defects on accelerating mass transport.

The diffusion coefficients of water and ions in this work are within the range of 10^−2^ Å^2^/ps, which matches well with the diffusion coefficient magnitude reported in asphalt–aggregate interface MD studies [32,33]. The relative increase trend induced by defects is also consistent with relevant literature, verifying the rationality of our diffusion results.

#### 3.3.2. Hydrogen Bond Analysis

**Figure 13:**
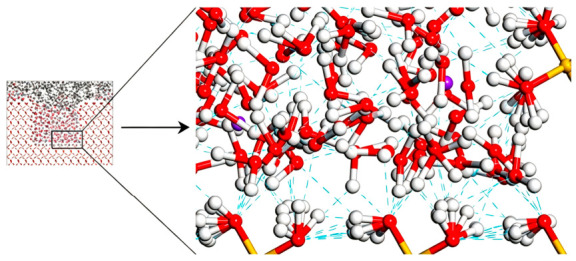
Schematic diagram of hydrogen bonds in the interface model.

To enhance understanding of moisture’s influence on adhesion properties at interfaces, this study investigates the role of hydrogen bonds within the framework of electrostatic interaction theory. A hydrogen bond represents a distinctive type of intermolecular or intramolecular interaction, occurring between a hydrogen atom (X-H) covalently bonded to an electronegative donor atom (such as O, N, or F) and a small-radius electronegative acceptor atom (Y, typically also O, N, or F). In this context, the hydrogen atom serves as a bridge, linking two electronegative atoms. Although hydrogen bonds are not classified as chemical bonds—since they do not involve electron sharing or transfer—they exhibit stronger interactions than van der Waals forces, yet are weaker than conventional chemical bonds. Notably, the strength of a hydrogen bond escalates significantly as the distance between the receptor atom and the hydrogen atom diminishes. In this investigation, the number of hydrogen bonds is determined through the average of multiple frame trajectories. Specifically, hydrogen bonds with lengths less than 2.2 Å and angles exceeding 90° between the aggregate and solution were quantified using a dedicated script, as illustrated in Figure 13. The count of hydrogen bonds correlates with the quantity of water molecules on the aggregate surface. An interaction force of zero between the asphalt and the aggregate signifies that water molecules envelop the aggregate surface, leading to a re-stabilization of hydrogen bond interactions between the water molecules and the aggregate. Data collection spanning at least 600 ps is conducted to compute the average number of hydrogen bonds present between the solution and the aggregate.

With the continuous infiltration of chloride ions and water molecules at the defective interface, the intrinsic hydrogen bonding between asphalt and aggregate was gradually weakened, while competitive hydrogen bonding between asphalt and water increased significantly. This substitution effect directly reveals the microscopic mechanism of water-induced interfacial stripping of asphalt–aggregate systems under salt solution immersion.

**Figure 14:**
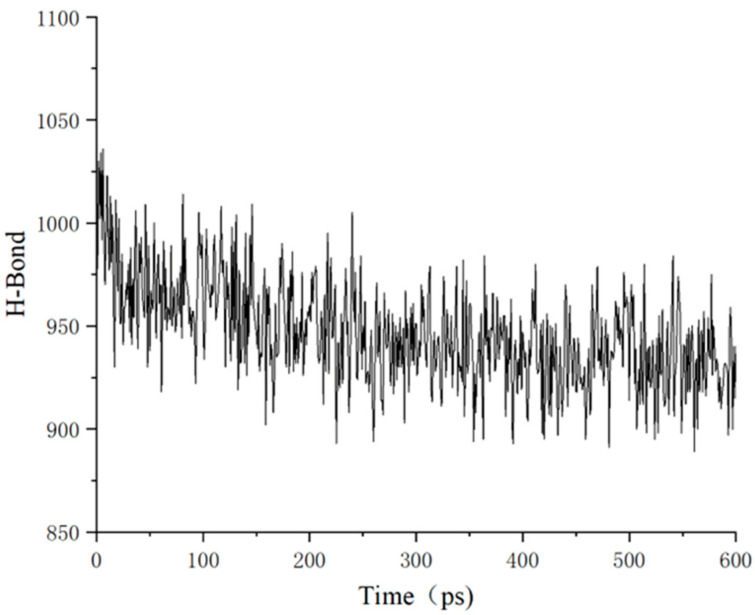
Graph of changes in the number of hydrogen bonds.

As illustrated in Figure 14, the number of hydrogen bonds exhibits a distinct fluctuating pattern over time. During the initial phase (0–50 ps), the count remains relatively high, oscillating between approximately 950 and 1050, which suggests strong intermolecular interactions and a stable system. However, as time progresses, the number of hydrogen bonds gradually decreases. By the middle and late stages (around 200 ps), the count stabilizes, and the range of fluctuations diminishes. This behavior indicates a gradual weakening of intermolecular interactions, with an increase in the breaking of hydrogen bonds and a corresponding trend toward increased molecular mobility and diffusion. Ultimately, the number of hydrogen bonds reaches a steady state around 950, signifying that the system has attained a relatively dynamic equilibrium. This observed trend aligns with the previously discussed influence of defects on diffusion behavior. The presence of defects facilitates the breaking of hydrogen bonds and accelerates the molecular diffusion process.

It should be emphasized that the hydrogen bond energy listed in Table 2 remains nearly constant under different defect sizes. This is because the hydrogen bond data in Table 2 mainly reflects the hydrogen bond interaction between the aggregate surface and solution, which is stable after dynamic equilibrium. The weakening of interfacial adhesion mainly comes from the reduction in van der Waals and electrostatic interactions, rather than the change of aggregate-solution hydrogen bonds. Therefore, the stable hydrogen bond energy, the decreased adhesion energy and the accelerated molecular diffusion are consistent in mechanism.

### 3.4. Effect of Chloride Salt Concentration

Varying concentrations of chloride salts induce significant alterations in the microtopography of the asphalt surface, which subsequently affect the interfacial adhesion properties between asphalt and aggregate. Consequently, this section examines the impact of different chloride salt concentrations on these interfacial adhesion characteristics. Utilizing an interface model of aggregate with defects measuring 20 Å as a case study, we analyze how chloride salt concentration influences interfacial adhesion energy. For this investigation, sodium chloride solutions with concentrations of 0%, 5%, 10%, and 15% have been selected.

**Figure 15:**
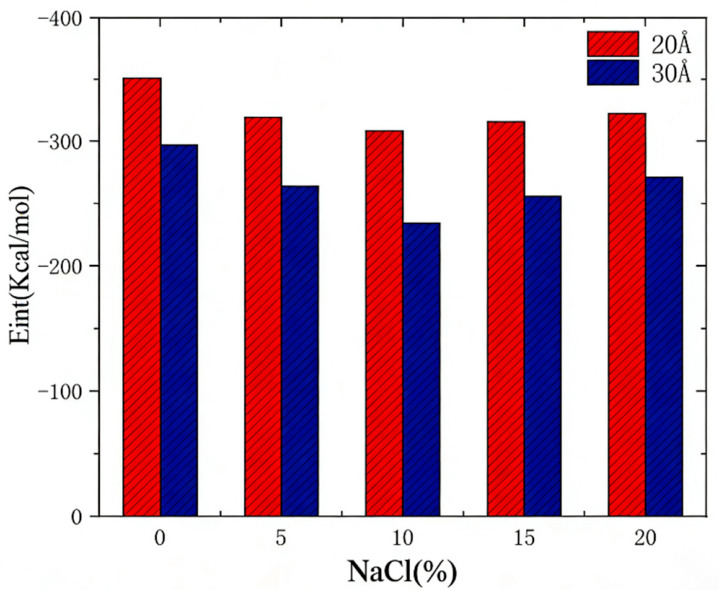
Interfacial adhesion energy variation with NaCl concentration (0–20%), showing a 15% decrease at high salt concentration.

An increase in NaCl concentration significantly impacts interfacial adhesion energy. As illustrated in Figure 15, higher NaCl concentrations enable ions in the solution to effectively shield the electrostatic interactions at the interface, leading to an increase in adhesion energy while simultaneously weakening the overall adhesion. Furthermore, this effect is more pronounced in the presence of larger defects (e.g., 30 Å). Larger defect areas are more prone to ion adsorption from the solution, which further diminishes the interaction forces at the interface. Consequently, it can be concluded that elevated NaCl concentrations reduce the adhesion between silica and bitumen, particularly in scenarios where larger surface defects are present. This conclusion is consistent with the research results of chloride erosion on asphalt–aggregate interface in the previous literature [1,2,5,22,23], which verifies the reliability of the simulation data in this study. As the NaCl concentration increases from 0% to 20%, the adhesion energy decreases by about 15%, indicating that higher salt concentration further weakens the interface bonding.

### 3.5. Effect of Temperature

**Figure 16:**
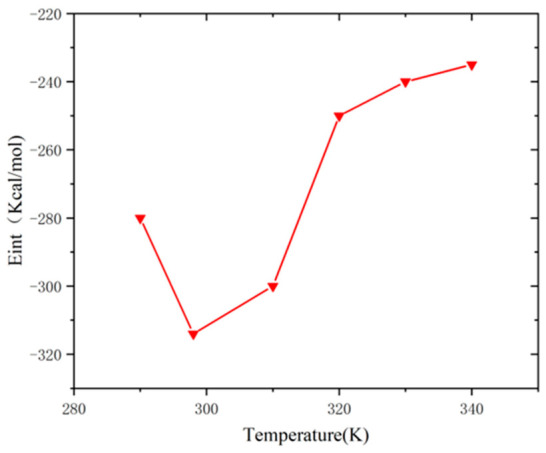
Interfacial adhesion energy evolution with temperature (290–340 K), showing a 28% reduction at elevated temperature.

**Figure 17:**
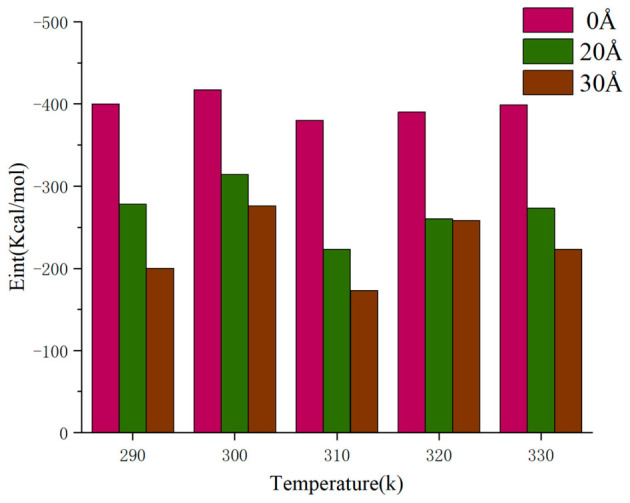
Relationship between adhesion energy and temperature with different defects.

Asphalt is a quintessential temperature-sensitive material, and its viscoelastic properties, including viscosity, are significantly influenced by temperature. Consequently, it is imperative to consider temperature’s effects when investigating the interfacial adhesion characteristics. To assess the impact of temperature on interfacial adhesion energy, we calculated the adhesion energy of the asphalt–aggregate interface model at six distinct temperatures: 290 K, 298 K, 310 K, 320 K, 330 K, and 340 K. Figure 16 illustrates the adhesion energy–temperature curve for the asphalt–aggregate interface model. Notably, the adhesion energy exhibits fluctuations with increasing temperature, with the absolute value peaking at 298 K, indicating optimal adhesion performance among the tested temperatures. The variance between the minimum and maximum adhesion energies is approximately 25%. At lower temperatures, the molecular mobility of asphalt is comparatively restricted, resulting in stronger van der Waals forces and hydrogen bonds among the molecules, thereby enhancing adhesion performance [35]. Low temperatures restrict the movement of asphalt molecules, leading to increased viscoelasticity and viscosity, which subsequently improves adhesion to the aggregate. The interfacial adhesion energy shows a continuous decreasing trend with increasing temperature, and 298 K is not a physical critical point. With increasing temperature, the thermal motion of asphalt molecules is enhanced continuously, which weakens intermolecular interactions and interfacial adhesion. Although asphalt molecules exhibit a certain degree of fluidity at this stage, it remains insufficient for effective adhesion. As temperature continues to rise, the movement of asphalt molecules intensifies [36]. While intermolecular adhesion forces further decline, the enhanced mobility of asphalt molecules allows for better accommodation of the irregularities on the aggregate surface, resulting in a fluctuating adhesion energy profile [37,38]. Figure 17 demonstrates that the presence of defects exacerbates this phenomenon. This temperature dependence of interface adhesion is consistent with the law of asphalt materials reported in [35,36,37,38], which further explains the weakening mechanism of interface under high temperature environment. With temperature rising from 290 K to 340 K, the adhesion energy decreases by approximately 28%, which quantifies the significant degradation effect of high temperature on interfacial adhesion.

The density of asphalt decreases gradually with increasing temperature, and the radial distribution function (RDF) indicates weakened molecular packing and reduced intermolecular correlation. The kinetic energy of molecules increases monotonically with temperature, which enhances molecular mobility and weakens interfacial adhesion. Each system is sufficiently equilibrated before data collection, and the potential energy, temperature, and density are stable. The Andersen thermostat is used with moderate coupling strength, which does not cause obvious dynamic perturbation or artificial artifacts in the simulation results.

The decreasing trend of interfacial adhesion energy with increasing temperature is consistent with the thermal response law observed in previous MD simulations of asphalt materials [35,37,38]. The reduction amplitude of approximately 28% in the tested temperature range is comparable to reported values, indicating that our thermal-dependent results are realistic and reasonable.

It should be emphasized that the above results are based on idealized molecular dynamics models, which cannot fully reflect the complex service environment, mechanical load, and long-term aging conditions of real asphalt pavements. Therefore, the conclusions can only reveal the microscopic mechanism and qualitative trend, rather than being directly extrapolated to macroscopic pavement performance and actual engineering durability.

## 4. Conclusions

This study reveals the adhesion characteristics of the asphalt–SiO_2_ aggregate interface under diverse environmental conditions. Surface defects introduce additional local adsorption sites by elevating surface roughness, thus enhancing local interfacial adhesion; nevertheless, overlarge defects disrupt the ordered surface structure, weaken effective interfacial interactions, and consequently reduce the absolute value of the overall interfacial adhesion energy, in which van der Waals forces act as the dominant contribution to interfacial adhesion, accompanied by secondary roles of hydrogen bonding and electrostatic interactions. Under the intrusion of chloride solution, NaCl primarily compromises interfacial adhesion via ion aggregation and electrostatic shielding effects, and elevated chloride concentrations tend to accelerate the degradation of both physical interfacial structure and adhesive bonding. In defective systems, the diffusion rates of water molecules, Cl^−^, and Na^+^ are notably facilitated, which further expedites the erosion process of the interface. Within the investigated temperature interval, the interfacial adhesion exhibits a distinct temperature dependence, with high temperatures exacerbating interfacial damage, while surface defects act to amplify the detrimental impacts of environmental erosion on the interface.

These findings emphasize the importance of considering surface defects and environmental factors in designing more durable asphalt materials, particularly in coastal and de-icing areas. The study also suggests opportunities for developing treatments to mitigate the adverse effects of defects and environmental stresses. In summary, understanding the interaction between defects, environmental conditions, and adhesion is crucial for improving the durability of asphalt–aggregate interfaces. Future work will extend the model to consider defect depth, slit-type microcracks, and quantitative roughness statistics to further improve the agreement with real aggregate microstructures.

There are certain computational limitations in this molecular dynamics model that should be clearly acknowledged. First, the simulation is limited to the nanoscale and short time scale (picoseconds), which is far from the actual service time and macroscopic length scale of real pavement engineering. Second, external mechanical loading, traffic fatigue, and long-term field aging conditions are not included in the current model. Third, the asphalt composition and interfacial environment are appropriately simplified, while real pavement systems involve more complex components and service conditions. Therefore, the simulation results can only reveal the microscopic adhesion mechanism and qualitative evolution trend, and cannot be directly extrapolated to quantitatively predict the macroscopic durability or field performance of actual pavements. All conclusions are for mechanism analysis and theoretical reference rather than direct quantitative engineering prediction.

## Figures and Tables

**Table 1:** Table of components of asphalt.

Component	Numerator	Molecular Weight	Number of Molecules	Mass Fraction
Resin	Asphaltene Phenol As1	575	1	10.08
	Asphaltene pyrrole	888.5	1	
	Asphaltene thiophene	707	2	
Asphaltenes	Benzobisbenzothiophene	290.4	21	31.75
	Pyridinohopane	503.9	2	
	Quinolinohopane	554.0	1	
	Thioisorenieratane	573.1	1	
Aromatic	DOCHN	414.8	2	48.65
	PHPN	406.8	17	
Saturate	Hopanesa1	464.8	15	9.52
	Squalane	483.0	3	

**Table 2:** Energy components of adhesion energy.

Defect	Energy Component	Average Energy (Kcal/mol)
0 Å	*E*_int_	−417.0695403
	*E*_No-Bond_	−417.0695403
	*E*_elec_	−367.8800738
	*E*_vdw_	−38.91904906
	*E*_H-Bond_	−10.27041739
20 Å	*E*_int_	−314.9757955
	*E*_No-Bond_	−314.9757955
	*E*_elec_	−281.5386203
	*E*_vdw_	−23.16675783
	*E*_H-Bond_	−10.27041739
30 Å	*E*_int_	−276.9986903
	*E*_No-Bond_	−276.9986903
	*E*_elec_	−235.4313858
	*E*_vdw_	−31.29688712
	*E*_H-Bond_	−10.27041739

**Table 3:** Diffusion coefficient of components.

Defect	H_2_O	Cl^−^	Na^+^
0 Å	0.05614	0.02303	0.01689
20 Å	0.06675	0.05242	0.04671
30 Å	0.09507	0.07285	0.06021

## Data Availability

The original contributions presented in this study are included in the article. Further inquiries can be directed to the corresponding author.
